# The Lost Pipe

**Published:** 1869-10

**Authors:** 


### THE LOST PIPE.

He was an old man and very sick; an inveterate smoker, he seemed only happy when behind a pipe. In apparent health he was stricken with paralysis, the physician thought from excessive smoking. He partially recovered. His son-in-law said to him one day, "Your pecuniary interests are large, are scattered and unsettled, you are liable any day to have a repitition of this late attack; it would be well for you to adjust your business, and above all to make a suitable preparation for the untried future." He smoked on and did nothing. A few days later he lost all sense but that of sight. He could not speak, nor move a finger or a limb. If he could hear or taste or smell, there were no means of ascertaining the fact. He could not communicate his wants in any way. And when there was an expression of countenance indicating that something was wanting, the only resource was to show him first one thing, then endeavor by watching the expression of his countenance to ascertain his wishes. On one occasion several hours had been spent in showing or touching everything that was visible in the room, but disappointment rested on his features; at last some one thought of his pipe, and on the instant of its presentation an almost seraphic gladness shot out from his glittering eyes; it was lighted and the stem introduced between his lips; the next instant he seemed to be in excruciating agony, only to be relieved by breaking into tears; he discovered that he had not the power to draw a single smoke; for several days he sobbed like an almost broken-hearted child, and then sunk into the sleep which knows no waking.

